# Combined kinetic analysis of SARS-CoV-2 RNAemia, N-antigenemia and virus-specific antibodies in critically ill adult COVID-19 patients

**DOI:** 10.1038/s41598-022-12461-5

**Published:** 2022-05-18

**Authors:** Rosa Costa, Juan Alberola, Beatriz Olea, Roberto Gozalbo-Rovira, Estela Giménez, Enric Cuevas-Ferrando, Ignacio Torres, Eliseo Albert, Nieves Carbonell, José Ferreres, Gloria Sánchez, Jesús Rodríguez-Díaz, María Luisa Blasco, David Navarro

**Affiliations:** 1 Microbiology Service, Clinic University Hospital, INCLIVA Health Research Institute, Av. Blasco Ibáñez 17, 46010 Valencia, Spain; 2 Department of Microbiology, School of Medicine, University of Valencia, Valencia, Spain; 3 Department of Preservation and Food Safety Technologies, Institute of Agrochemistry and Food Technology, IATA-CSIC, Valencia, Spain; 4 Medical Intensive Care Unit, Clinic University Hospital, INCLIVA Health Research Institute, Valencia, Spain

**Keywords:** Immunology, Microbiology

## Abstract

Combined kinetic analysis of plasma SARS-CoV-2 RNAemia, Nucleocapsid (N)-antigenemia and virus-specific antibodies may help ascertain the role of antibodies in preventing virus dissemination in COVID-19 patients. We performed this analysis in a cohort of 71 consecutive critically ill COVID-19 patients (49 male; median age, 65 years) using RT-PCR assay, lateral flow immunochromatography method and receptor binding domain (RBD) and N-based immunoassays. A total of 338 plasma specimens collected at a median of 12 days after symptoms onset were available for analyses. SARS-CoV-2 RNAemia and N-antigenemia were detected in 37 and 43 specimens from 26 (36.5%) and 30 (42.2%) patients, respectively. Free RNA was the main biological form of SARS-CoV-2 found in plasma. The detection rate for both viral components was associated with viral load at the upper respiratory tract. Median time to SARS-CoV-2-RBD antibody detection was 14 days (range, 4–38) from onset of symptoms. Decreasing antibody levels were observed in parallel to increasing levels of both RNAemia and N-antigenemia, yet overall a fairly modest inverse correlation (Rho = −0.35; *P* < 0.001) was seen between virus RNAemia and SARS-CoV-2-RBD antibody levels. The data cast doubts on a major involvement of antibodies in virus clearance from the bloodstream within the timeframe examined.

## Introduction

SARS-CoV-2 seemingly replicates in a wide variety of organs and tissues, which contributes towards explaining the multisystemic nature of COVID-19^1^. A plausible pathogenic scenario is that following initial replication in the respiratory tract, SARS-CoV-2 surpasses the epithelial barrier and disseminates through the bloodstream, reaching extrapulmonary sites^2^. Supporting this view, apparently intact virions have been identified in plasma pellets from COVID-19 patients using electron tomography and immunostaining^3^; however, SARS-CoV-2 has never been cultivated from plasma specimens^4^. Furthermore, virus RNAemia, antigenemia (nucleocapsid-N- or Spike-S) or both are frequently detected in COVID-19 patients, at a rate modulated by the sensitivity of the analytical methods employed and notably by disease severity^5–18^. In this latter regard, it has been shown that detection of either of these viral components within the first two weeks after SARS-CoV-2 infection diagnosis is associated with intensive care unit (ICU) admission, need for invasive mechanical ventilation, multiple organ failure and mortality rate^5–18^.

Functional antibodies, especially those displaying virus neutralization capacity, may contribute to SARS-CoV-2 clearance from the bloodstream^19^, thus minimizing virus dissemination. If this assumption holds true, circulating antibody levels relatively early after infection may serve as a surrogate marker for poor clinical outcomes. In this setting, contradictory data have been published on the relationship between serum levels of antibodies with either neutralizing or uncharacterized functions and the magnitude of SARS-CoV-2 RNAemia and N-antigenemia^3,4,10,13,14,16,17,20–23^. To shed light in this issue, here we conducted a combined kinetic analysis of SARS-CoV-2 RNAemia, N-antigenemia and virus-specific antibodies in sequential plasma specimens from a relatively homogeneous cohort of critically ill adult COVID-19 patients. To enhance data interpretation, we also sought to elucidate the biological form of SARS-CoV-2 present in blood.

## Material and methods

### Patients and specimens

In this prospective observational study, 71 consecutive critically ill COVID-19 patients (49 male and 22 female; median age, 65 years; range, 21–80 years) were enrolled between October 2020 and February 2021 (Table 1). These patients belonged to a previously reported cohort^6,18^, of which two participants could not be included herein due to the lack of data on SARS-CoV-2 antibodies. Plasma specimens were obtained on a weekly basis after ICU admission, when possible, by centrifugation of whole blood EDTA tubes, cryopreserved and retrieved for the analyses described below. Medical history and laboratory data were prospectively recorded. The current study was approved by the Research Ethics Committee of Hospital Clínico Universitario INCLIVA (May, 2020). All experiments were performed in accordance with relevant local guidelines and regulations. Informed consent was obtained from all participants, either on the hospital ward or at the time of ICU admission.

**Table 1:** Clinical characteristics of the study population at Intensive Care Unit admission.

Variable	no. (%)
**Gender**	
Male	49 (69.1)
Female	22 (30.9)
**Acute physiology and chronic health evaluation (APACHE) II score**	
< 10	14 (19.7)
10–14	26 (36.6)
15–29	31 (43.7)
**Comorbidities**	
Diabetes mellitus	17 (23.9)
Asthma/chronic lung disease	11 (15.5)
Hypertension	32 (45.0)
Obesity	38 (53.5)
Chronic heart disease	9 (12.6)
Vascular disease	7 (9.8)
Cancer	3 (4.2)
Hematologic disease	3 (4.2)
**Number of comorbidity conditions**	
One	22 (31.0)
Two or more	32 (45.0)
None	17 (24.0)
**Oxygenation and ventilator support**	
Invasive mechanical ventilation	63 (88.7)
PiO_2_/FiO_2_ < 150 mmHg	57 (80.2)
Acute kidney disfunction	17 (23.9)
**Antiviral or anti-inflammatory treatment**	
Remdesivir	15 (21.1)
Corticosteroids	70 (98.5)
Tocilizumab	27 (38.0)

### Detection of SARS-CoV-2 RNA in nasopharyngeal specimens and plasma by RT-PCR

Nasopharyngeal specimens (NP) collected in 3 ml of Universal Transport Medium (UTM, Becton Dickinson, Sparks, MD, USA) were analyzed by RT-PCR within 24 h of receipt. The TaqPath COVID-19 Combo Kit (Thermo Fisher Scientific, MS, USA) was used, following RNA extraction carried out by using the Applied Biosystems™ MagMAX™ Viral/Pathogen II Nucleic Acid Isolation Kits coupled with the Thermo Scientific™ KingFisher Flex automated extraction instrument. Nucleic acid was extracted from plasma (400 µl) with the Abbott mSample Preparation System DNA kit (Abbott Molecular, Des Plaines, IL, USA) on the Abbott m2000sp platform (Abbott Molecular), while SARS-CoV-2 RNA amplification was carried out using the Abbott Real*Ti*me SARS-CoV-2 assay on the m2000rt platform, as previously described^6^. The limit of detection of the assay for plasma was found to be approximately 100 copies/ml (95% CI)^6^. SARS-CoV-2 viral loads in both specimen types were estimated using the AMPLIRUN^®^ TOTAL SARS-CoV-2 RNA Control (Vircell SA, Granada, Spain)^6^, and reported as copies/ml throughout the study.

### SARS-CoV-2 RNA-viability RT-qPCR assay

Using a previously published protocol^24^, plasma specimens were diluted 1/10 in PBS, pretreated or not at high temperature (95 °C, 10 min) then treated with 5 mM of platinum chloride (PtCl_4_) for 30 min at room temperature in DNA LoBind tubes (Eppendorf, Germany) in an orbital shaker (150 rpm). Viral RNA was then extracted using Maxwell^®^ RSC 16 instrument and Maxwell RSC Pure Food GMO and authentication kit (Promega, Spain) and amplified by RT-qPCR targeting the N gene (N1 sequence). SARS-CoV-2 RT-qPCR negative plasma specimens spiked with heat-inactivated SARS-CoV-2 (approximately, 10^5^ gc/mL) were used as controls. Under these conditions, lack of viral RNA amplification in non-pretreated plasma following PtCl_4_ treatment was interpreted as compatible with presence of free viral RNA (absence of viable virus) in the specimen.

### Detection of SARS-CoV-2 N protein in plasma

The lateral flow immunochromatography (LFIC) device CLINITEST^Ⓡ^ Rapid COVID-19 Antigen Test (Siemens, Healthineers, Erlangen, Germany) was used for detection and grading of SARS-CoV-2 N-antigenemia in plasma specimens, as previously described^18^. The analytical sensitivity of the assay is around 50 pg/ml^18^. N-antigen line intensity was scored visually by five researchers in the group (RC, BO, EG, IT and EA) using a 3‐level scale: 0, negative result; 1 + , intensity of test band lower than control band and 2 + , intensity of test band equal to or greater than control line^18^. When there was no consensus on degree of reactivity, the one designated by the majority of observers was taken as the final one.

### Antibody detection immunoassays

SARS-CoV-2-RBD-reactive IgG antibodies were quantitated by an in-house-developed immunoassay using recombinant RBD produced in Sf9 insect cells, following a previously published protocol^25^. The chemiluminescent Abbott Alinity SARS-CoV-2 anti-nucleocapsid protein IgG assay was used to measure (semiquantitative) SARS-CoV-2-N-reactive IgGs.

### Statistical methods

Frequency comparisons for categorical variables were performed using Fisher’s exact test. Differences between medians were compared using the Mann–Whitney U test. Correlations between variables of interest were calculated by the Spearman Rank test. Two-sided exact *P* values were reported. A *P* value < 0.05 was considered statistically significant. The analyses were performed using SPSS version 20.0 (SPSS, Chicago, IL, USA).

## Results

### Clinical characteristics of patients

Patients were admitted to ICU at a median of 8 days (range, 2–25) after onset of symptoms. Most patients eventually required mechanical ventilation (88.7%). Median time of ICU stay was 19 days (range, 2–67). All patients but one were treated with corticosteroids, whereas remdesivir and tocilizumab were used in 21% and 38% of patients, respectively.

### Detection of SARS-CoV-2 RNAemia and N-antigenemia in the cohort

**Figure 1:**
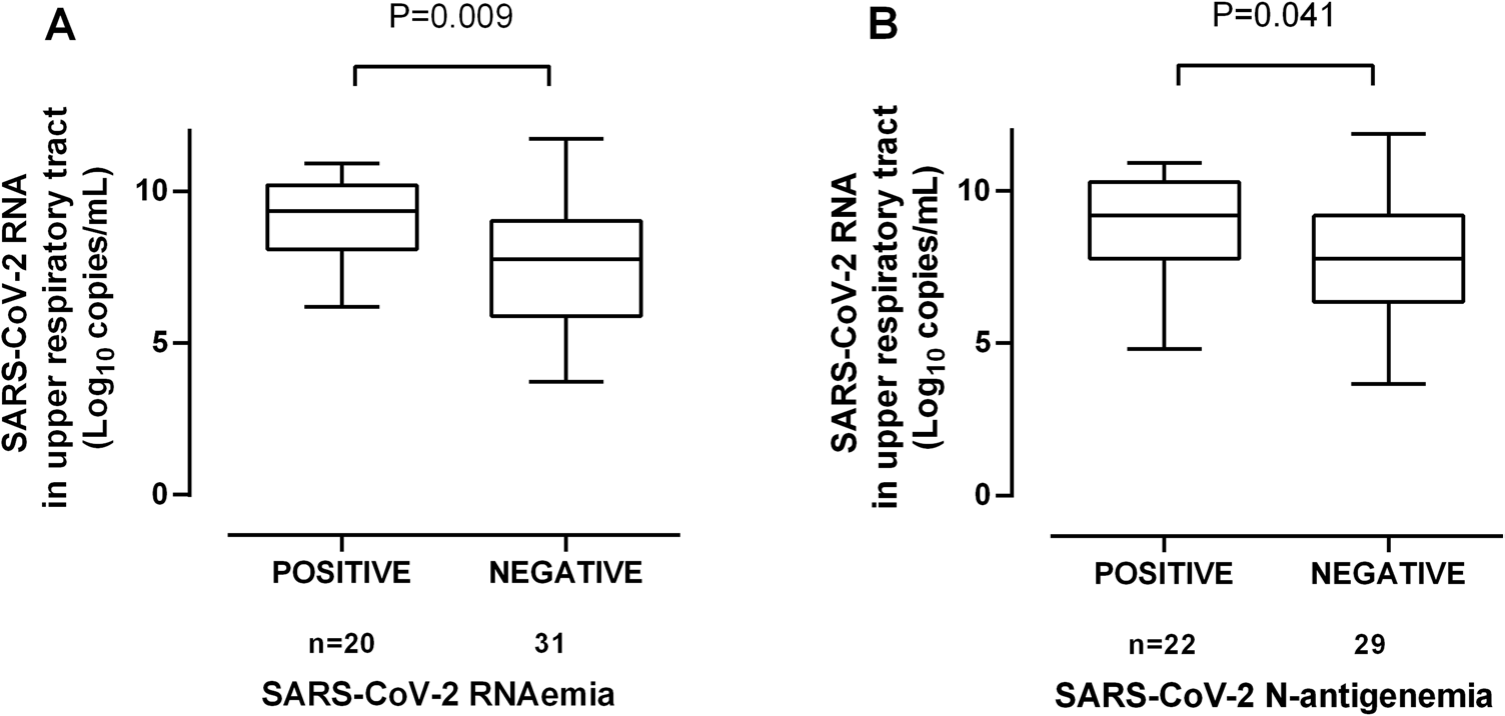
Box-plot depicting SARS-CoV-2 RNA load (in copies/ml) in nasopharyngeal specimens at the time of molecular diagnosis of SARS-CoV-2 infection in critically ill patients with or without subsequent virus RNAemia (**A**) or N-antigenemia (**B**). *P* values are shown for comparisons.

A total of 338 plasma specimens from 71 patients (median, 4 samples/patient; range, 1–16) collected following ICU admission were available for analyses. Overall, the first plasma specimen in these patients was drawn at a median of 12 days (range, 3–38 days) after symptoms onset. SARS-CoV-2 RNAemia and N-antigenemia were detected in 37 and 43 specimens from 26 (36.5%) and 30 (42.2%) patients, respectively. Since the extent of virus replication in the upper respiratory tract (URT) may impact on the access of intact virus particles or viral products to the bloodstream, we investigated whether SARS-CoV-2 RNA load in NPs early following infection (median of 4 days after symptoms onset; range, 1–11) was associated with the likelihood of detecting viral RNAemia and N-antigenemia. SARS-CoV-2 RNA load estimates in NP specimens at the time of diagnosis were available for 51 of 71, of whom 20 and 22 had detectable viral RNAemia and N-antigenemia, respectively, during follow-up. In the remaining 20 patients either diagnosis of SARS-CoV-2 infection was made by using N-antigen detection LFIC tests or RT-PCR C_T_ values were not available. As shown in Fig. 1, patients developing virus RNAemia and N-antigenemia exhibited significantly higher viral SARS-CoV-2 RNA load in NP (*P* = 0.009 and *P* = 0.04, respectively) at diagnosis. Moreover, a tentative positive correlation between NP viral loads and viral RNAemia levels was observed in first positive RT-PCR plasma specimens (Rho = 0.42; *P* = 0.1). Notably, neither remdesivir nor tocilizumab treatment had an impact on the rate of positive SARS-CoV-2 RNAemia (*P* = 0.57 and 0.60, respectively) or N-antigenemia (*P* = 0.58 and *P* = 0.41, respectively).

### Dynamics of SARS-CoV-2 RNAemia, N-antigenemia and virus-specific antibodies

Time to first positive viral RNAemia and N-antigenemia result was 10 days (range, 3–32) and 9 days (range, 3–29), respectively, since the onset of symptoms. Both virus components were first detected within 16 days after symptoms appearance in all but one patient. No sample obtained after day 32 had detectable viral RNAemia or N-antigenemia.

SARS-CoV-2-RBD antibodies were eventually detectable in 67 out of the 71 patients, with a median time to first detection of 14 days (range, 4–38) since onset of symptoms. Of the 4 patients who did not develop measurable antibodies, 2 had detectable viral RNAemia and N-antigenemia. Time to first detection of antibodies was comparable for patients with or without viral RNAemia (*P* = 0.21) and N-antigenemia (*P* = 0.1) and was not significantly correlated with initial viral load in NP (Rho = 0.229; *P* = 0.11). The time course of detection of SARS-CoV-2 RNAemia, N-antigenemia and RBD-IgG reactive antibodies is shown in Table 2. Note that the number of plasma specimens in which viral RNAemia or N-antigenemia and anti-RBD IgG antibodies were co-detected was rather small (25 and 24 samples of 338, respectively).

**Table 2:** Detection of SARS-CoV-2 RNAemia, N-antigenemia and anti-RBD IgG stratified by time since COVID-19 symptoms onset in critically ill patients.

Combination of qualitative results for SARS-CoV-2 RNAemia or N-antigenemia and anti-RBD IgGs (total no. of specimens)	Time evolved since symptoms onset			
	Week 1. no. of specimens	Week 2. no. of specimens	Week 3. no. of specimens	≥ Week 4. no. of specimens
Detection of viral RNAemia and anti-RBD IgG (n = 25)	4	12	7	2
Detection of Detection of viral RNAemia and lack of anti-RBD IgG (n = 12)	5	5	1	1
Absence of viral RNAemia and detection of anti-RBD IgG (n = 272)	4	40	83	145
Absence of viral RNAemia and lack of anti-RBD IgG (n = 29)	2	9	4	14
Detection of viral N-antigenemia and anti-RBD IgG (n = 24)	6	16	0	2
Detection of viral N-antigenemia and lack of anti-RBD IgG (n = 19)	6	9	2	2
Absence of viral N-antigenemia and detection of anti-RBD IgG (n = 273)	2	36	90	145
Absence of viral N-antigenemia and lack of anti-RBD IgG (n = 22)	1	5	3	13

*RBD* receptor binding domain.

**Figure 2:**
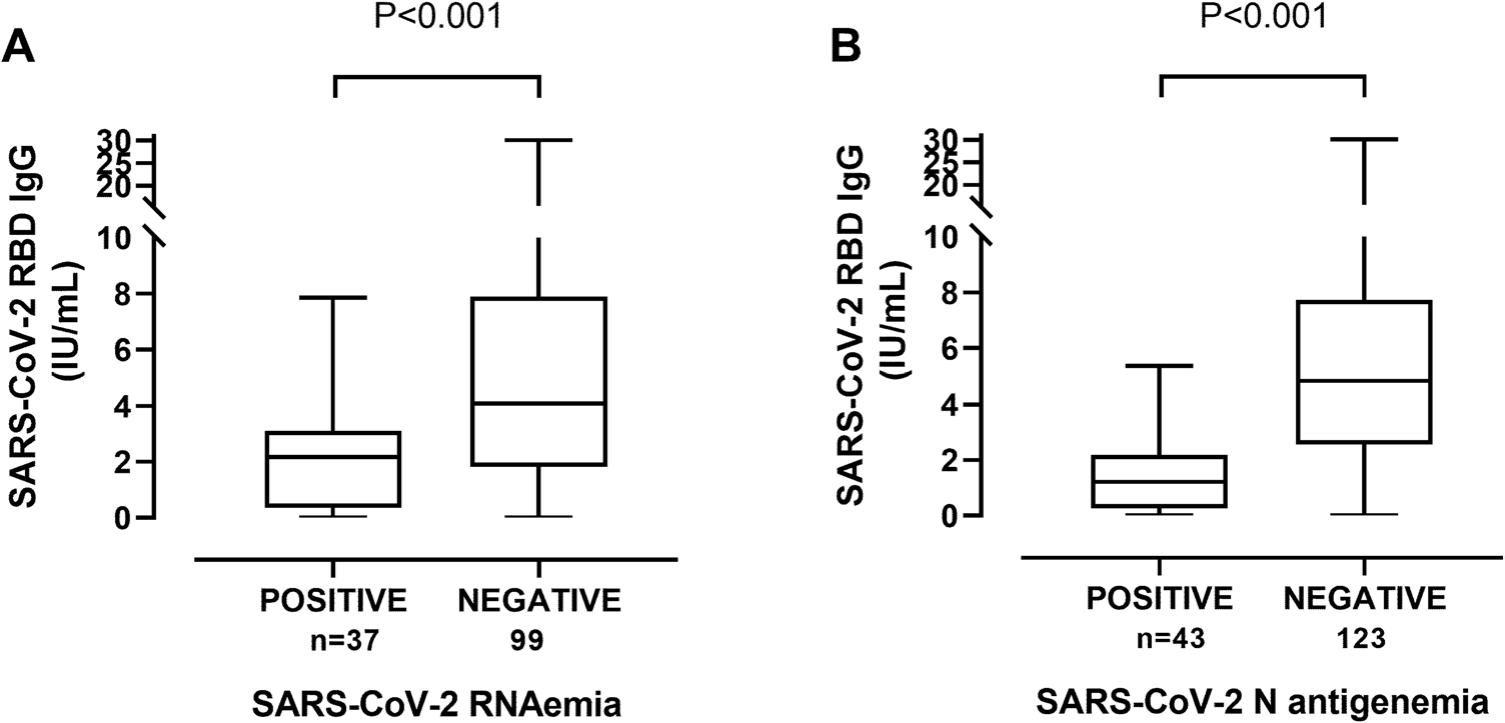
Box-plot depicting anti-SARS-CoV-2-S (RBD) antibody levels in plasma according to presence or absence of virus RNAemia (**A**) or N-antigenemia (**B**). *P* values are shown for comparison.

**Figure 3:**
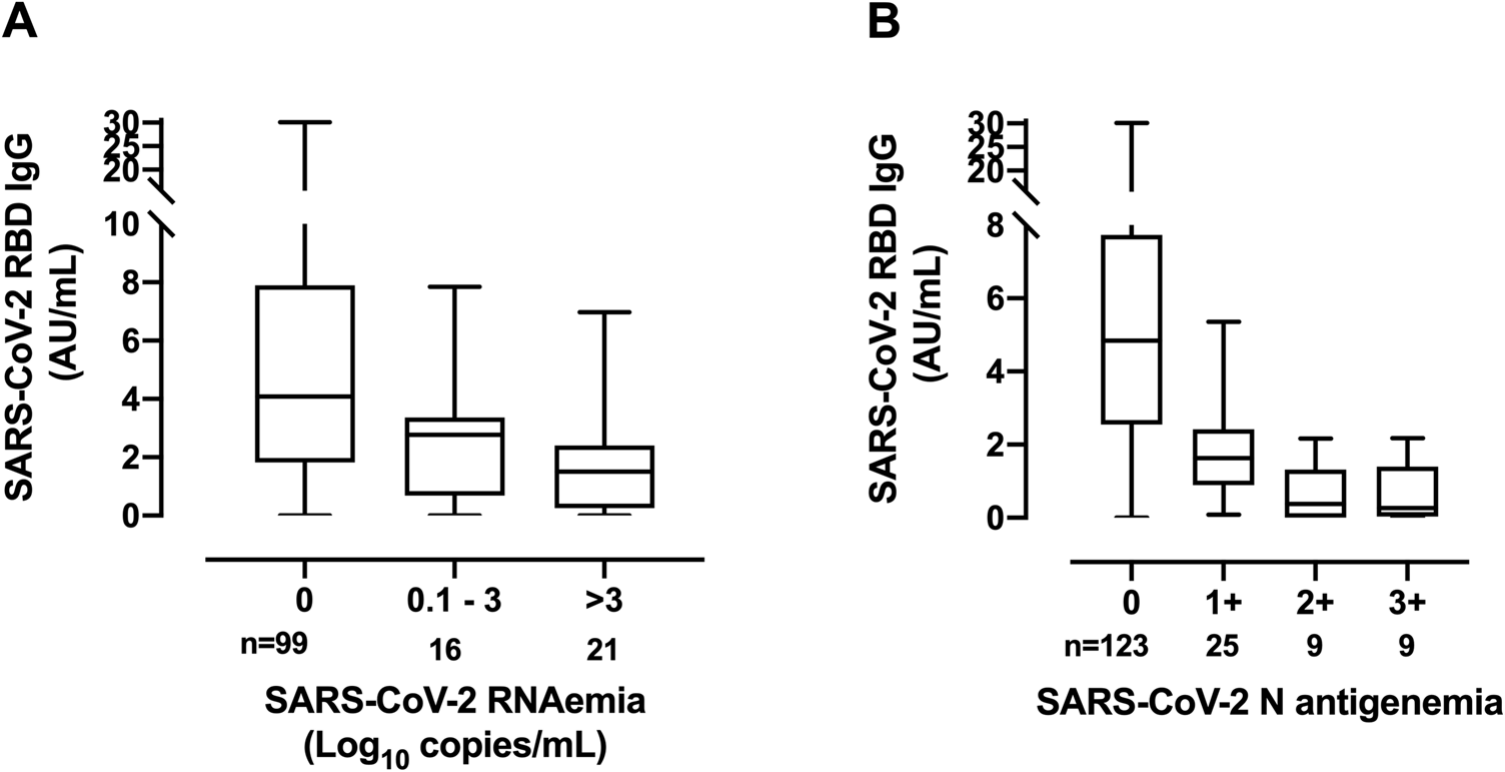
Box-plot depicting anti-SARS-CoV-2-S (RBD) antibody levels in plasma according to (**A**) the magnitude of virus RNAemia (in copies/ml) or (**B**) N-antigenemia (graded as described in the methods section).

**Figure 4:**
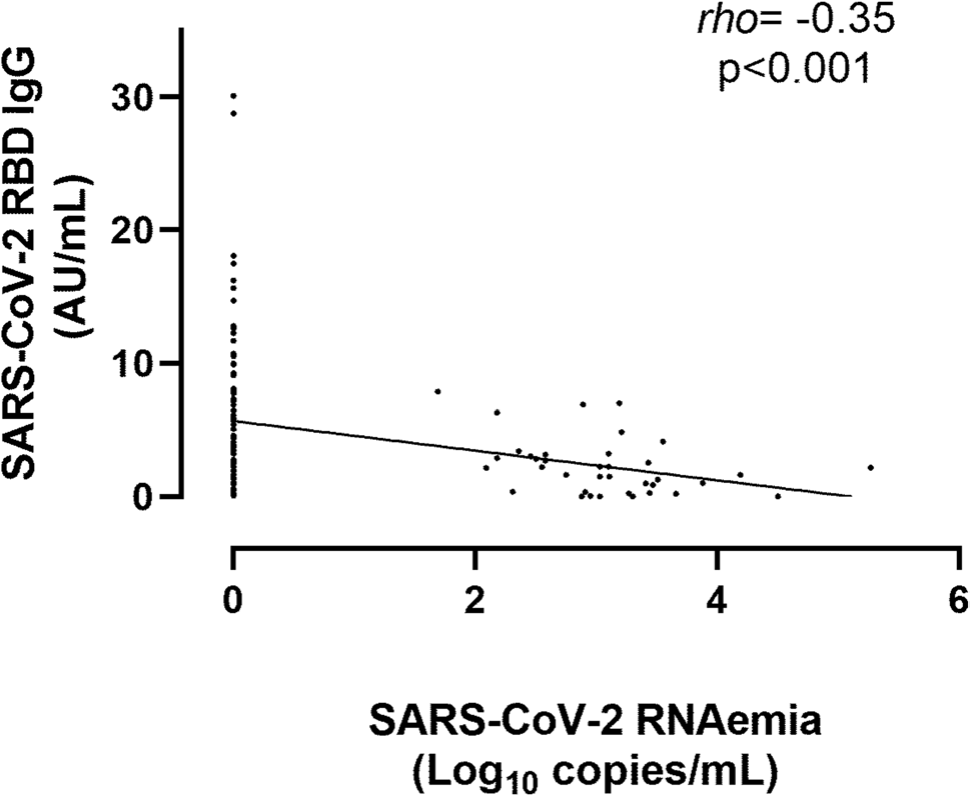
Overall correlation between anti-SARS-CoV-2-S (RBD) antibody and SARS-CoV-2 RNA levels in plasma. Rho and *P* values are shown.

Overall, specimens containing viral RNA or N protein had significantly lower serum SARS-CoV-2-RBD antibody levels (Fig. 2) than those in which these viral components were not detected. Moreover, decreasing antibody levels were observed in parallel to increasing levels of both RNAemia and N-antigenemia (Fig. 3). Yet overall, a fairly modest inverse correlation (Rho = −0.35; *P* < 0.001) was seen between virus RNAemia and SARS-CoV-2-RBD antibody levels (Fig. 4).

Data on SARS-CoV-2-N-reactive antibodies were only available for 50 plasma specimens, of which 16 tested positive by N-antigen LFIC assay. A trend (*P* = 0.16) towards higher N-antibody levels was seen for plasma testing negative by LFIC assay than in plasma testing positive.

### Characterization of the biological form of SARS-CoV-2 RNA in plasma

A total of seven plasma specimens from unique patients with detectable SARS-CoV-2 RNAemia (median load, 3.2 log10/ml; range, 3.0–3.8), drawn at a median of 9 days after symptoms onset (range, 3–12), were run on the SARS-CoV-2 RNA-viability RT-PCR assay. The data are shown in Table 3. The RT-PCR signal was completely abolished following PtCl_4_ treatment in 6 of the 7 non-pretreated (heated) specimens, whereas in the remaining one (specimen 2, obtained 3 days after symptoms onset) viral RNA was still detected, albeit at a lower level (approximately 1 log_10_). Treatment of heat-inactivated virus preparations with PtCl_4_ usually resulted in a threefold reduction in the level of viral RNA detected (not shown). The data thus suggested that viral RNA was likely in a non-capsidated, free state in the majority of plasma specimens subjected to analysis.

**Table 3:** RT-PCR viability assay performed with plasma from unique intensive care unit patients.

Patient number/day at which plasma was drawn after symptoms onset	Pre-treatment (95 ºC, 10 min)	PtCl_4_ (5 mM) treatment	RT-PCR N1 gene cycle threshold	
			Replicate 1	Replicate 2
1/5	No	No	36.5	36.4
		Yes	ND	ND
	Yes	No	36.4	36.4
		Yes	ND	ND
2/3	No	No	34.8	35.0
		Yes	37.3	37.1
	Yes	No	34.9	35.3
		Yes	ND	ND
3/7	No	No	34.5	34.7
		Yes	ND	ND
	Yes	No	34.9	35.3
		Yes	ND	ND
4/9	No	No	34.7	34.1
		Yes	ND	ND
	Yes	No	35.6	35.9
		Yes	ND	ND
5/12	No	No	34.7	35.8
		Yes	ND	ND
	Yes	No	35.8	35.8
		Yes	ND	ND
6/11	No	No	35.5	35.2
		Yes	ND	ND
	Yes	No	35.9	36.4
		Yes	ND	ND
7/11	No	No	36.5	37.7
		Yes	ND	ND
	Yes	No	36.6	ND
		Yes	ND	ND

*ND* not detected.

## Discussion

Occurrence of viral RNAemia and N-antigenemia early after SARS-CoV-2 infection has been associated with poor clinical outcomes, including increased mortality in critically ill COVID-19 patients^5–18^. The magnitude of viral RNAemia and N-antigenemia may reflect the extent to which SARS-CoV-2 is replicating in the respiratory tract, which may itself impact on the clinical course of infection^26^. In turn, SARS-CoV-2 specific antibodies may prevent or minimize infection of extrapulmonary sites by neutralizing free virions in the systemic compartment and contributing to virus clearance from bloodstream by immunocomplexed viral particles, which are then targeted for degradation by innate immune cells. Although mechanistically plausible, neither of the above assumptions have been clearly proven. To address these issues, we investigated the dynamics of SARS-CoV-2 RNAemia and N-antigenemia relative to that of SARS-CoV-2-specific antibodies, and the influence on these parameters of viral load in the URT at diagnosis, in a relatively homogeneous cohort of ICU patients. As previously reported^6,18^, viral RNAemia and N-antigenemia occurred frequently in our cohort (36.5% and 42.2% of patients respectively), were first detected relatively early after COVID-19 symptoms onset (within 2 weeks), and were not detectable beyond day 32 after symptoms onset. In our cohort, the likelihood of detecting either of these viral components in plasma from ICU patients was directly related to the magnitude of SARS-CoV-2 load in NP at diagnosis. A similar finding was reported by Le Hingrat et al.^16^, albeit in a mixed cohort in which ICU patients were underrepresented. Moreover, viral loads in NP and in first RT-PCR positive plasma specimens tended to correlate. Taken collectively, the data therefore point to a link between extent of virus replication in URT and virus burden in the blood compartment. Nevertheless, time to seroconversion was not correlated with viral load in NP. This is in contrast to data from a previously published study^27^, although differences in patient characteristics across cohorts and notably immunoassays used across the studies may help explain the discrepancy.

Passive transfer of SARS-CoV-2-S-reactive monoclonal antibodies has been shown to mediate virus clearance from the respiratory tract in both experimental models and humans^20,28,29^. Although virus-specific antibodies may plausibly contribute to SARS-CoV-2 clearance from the bloodstream, this assumption remains to be conclusively proven. In this context, demonstration of an inverse relationship between serum levels of SARS-CoV-2-reactive antibodies and the magnitude of SARS-CoV-2 RNAemia could indicate antibody involvement, provided that mature virus particles enter the blood compartment. Here, the frequency of detection of viral RNA or N protein in plasma was inversely related to serum SARS-CoV-2-RBD and anti-N antibody levels, respectively, and a trend towards a quantitative inverse association was observed, yet we found an at best modest (inverse) correlation between virus RNAemia and SARS-CoV-2-RBD antibody levels. Use of a semiquantitative assay precluded conducting a similar analysis for N-antigenemia, regarding which the data in the literature are contradictory. For example, in line with our findings, Martín-Vicente and colleagues^17^ reported that frequency of N-antigenemia early following ICU admission (24 h) was > 2.5 fold higher in the absence of anti-SARS-CoV-2 S antibodies than in those with detectable antibodies. In addition, levels of anti-S antibodies correlated inversely (albeit modestly) with viral RNA load in plasma (Rho = −0.45: *P* < 0.001). Other studies also found a higher rate of detection of either viral RNAemia N-antigenemia or both in the presence of low or undetectable anti-S or anti-N antibody levels in mixed cohorts^4,13,16^. In contrast, several studies failed to show an inverse correlation between neutralizing antibody titers and viral RNA levels in blood^3,14,23^; nevertheless, antibodies with functional activities other than virus neutralization may contribute to virus clearance from blood. Interpretation of the above data is not straightforward, due to marked differences across studies in terms of patient characteristics, particularly the analytical features of the immunoassays employed, type of specimen processed (sera or plasma), use of unique or sequential specimens, and timing of sample collection after symptoms onset. In addition, since immunoassays are only capable of detecting either antibodies in a free state or antigen–antibody immunocomplexes with available antibody binding sites^22^, elucidating the biological form of the virus present in blood (either infectious or defective genome viral particles, free viral components or both) may help to correctly interpret virus–antibody dynamics. In this respect, by using a RT-PCR viability assay we showed that within the timeframe examined, most viral RNA present in blood is likely in a non-capsidated state, indicating that the main source of viral products (RNA and proteins) in blood could be PANoptosis in the URT^22^; nonetheless, one out of seven plasma specimens yielded a RT-PCR viability profile compatible with presence of capsidated (protected) virus RNA. In this sense, apparently intact virus particles have been observed in plasma pellets using electron tomography and immunostaining^3^; of note, that latter specimen was obtained early after symptoms onset (3 days), suggesting that the presence of intact virions in the bloodstream may be restricted to a narrow window close to the time of contracting the infection.

The study has several limitations that must be acknowledged. First, its relatively small sample size, which precluded robust statistical subanalyses assessing the impact of demographics, clinical risk factors and use of different therapies on patient’s outcomes. Second, at ICU admission, most patients were at an advanced phase of SARS-CoV-2 infection, when the clinical picture is mainly related to the aberrant virus-triggered inflammatory response. Third, the use of analytical methods with suboptimal sensitivity for RNAemia and N-antigenemia detection, compared to droplet digital PCR and chemiluminescent assays, respectively^3,15,16^, and the lack of data on S-antigenemia. Moreover, functional characterization of antibodies, such evaluation of its virus neutralizing activity, was not done; yet plasma levels of antibodies binding the RBD strongly correlate with neutralizing antibody titers^30^. In contrast, our analysis of sequential specimens from patients could be considered a strength of the research.

In summary, the data presented herein support that the rates of detection of SARS-CoV-2 RNAemia and N-antigenemia, but not time to first anti-S-antibody detection, are modulated by the level of virus replication in the URT. Moreover, although viral RNAemia and N-antigenemia were more likely to be documented in patients with low than high anti-S or anti-N antibody levels, the relatively modest correlation between these levels and viral RNA loads in plasma specimens argue against major antibody involvement in virus clearance from the bloodstream, at least within the time window examined (median of 12 days after onset of symptoms), in which free virus components instead of intact virus particles appeared to be the main biological form of SARS-CoV-2 in blood. Whether a different scenario takes place earlier after infection is a possibility whose clinical implications warrant further research.

## Data Availability

The data that support the findings of this study are available from the corresponding author upon reasonable request.
